# Acridine and Its Derivatives: Synthesis, Biological, and Anticorrosion Properties

**DOI:** 10.3390/ma15217560

**Published:** 2022-10-28

**Authors:** Lipiar K. M. O. Goni, Mohammad A. Jafar Mazumder, Divya B. Tripathy, Mumtaz A. Quraishi

**Affiliations:** 1 Chemistry Department, King Fahd University of Petroleum & Minerals, Dhahran 31261, Saudi Arabia; 2 Interdisciplinary Research Center for Advanced Materials, King Fahd University of Petroleum & Minerals, Dhahran 31261, Saudi Arabia; 3 School of Basic and Applied Sciences, Galgotias University, Greater Noida 210310, India

**Keywords:** acridines, corrosion, metallic corrosion, corrosion inhibitors, adsorption, corrosion protection

## Abstract

The phenomenon of corrosion threatens metallic components, human safety, and the economy. Despite being eco-friendly and promising as a corrosion inhibitor, acridine has not been explored to its full potential. In this review, we have discussed multiple biological activities that acridines have been found to show in a bid to prove that they are environmentally benign and much less toxic than many inhibitors. Some synthetic routes to acridines and substituted acridines have also been discussed. Thereafter, a multitude of acridines and substituted acridines as corrosion inhibitors of different metals and alloys in various corrosive media have been highlighted. A short mechanistic insight into how acridine-based compounds function as corrosion inhibitors have also been included. We believe this review will generate an impression that there is still much to learn about previously reported acridines. In the wake of recent surges to find efficient and non-toxic corrosion inhibitors, acridines and their analogs could be an appropriate answer.

## 1. Introduction

The assault of corrosion on metallic structures has become an extremely widespread and persistent dilemma across the globe. In general, corrosion has been defined as the deterioration of metallic structures by reaction with the surrounding environment [1,2]. However, in a more technical sense, corrosion entails the movement of mass and charge across a metal/solution interface [3]. The phenomenon of corrosion ensues in the presence of four things: an anode, a cathode, a metallic path, and an electrolyte. Upon contact with an appropriate electrolyte, the anode part loses free electrons that travel through the electrolyte to reach the cathode. Those free electrons are consumed to produce molecular hydrogen in acidic media and hydroxyl ions in basic/neutral conditions contaminated with oxygen. There are acids (H_2_SO_4_, HCl, and HNO_3_) and bases (NaOH, CaCO_3_, NaHCO_3_) that commonly attack metallic structures. Besides, there is moisture/water (H_2_O), salts (NaCl), gases (formaldehyde, ammonia, sulfur-containing gases, etc.), and aggressive metal polishes as well that have also been found to be aggravating to metallic structures [4]. Metals have tremendous implications in the construction industry for building pipework, structural components, cladding materials, etc., owing to their outstanding strength and durability. Because of the ubiquitous nature of metallic elements, it is not surprising that they are always in contact with different corrosive scenarios, making corrosion damage prevalent and demanding so much attention [5].

Corrosion profoundly impacts human health and safety, environment, materials’ life span, and the economy. It has been reported that any country in the world spends around 1–5% of its gross national product (GNP) on corrosion [6]. The global cost of corrosion is so huge that a study done by the National Association of Corrosion Engineers (NACE) revealed that the global cost of corrosion for the year 2013 was 3.4% (equivalent to USD 2.5 trillion) of the global gross domestic product (GDP) [7]. A more detailed account of how corrosion impacted the economy and claimed human lives in the past can be found in the previously published literature [8]. Corrosion inhibition [9,10,11], anodic protection [12], cathodic protection [13,14], alloying [15,16], and coatings [17,18] are some of the most widely used approaches to combat corrosion. However, corrosion inhibitors (CIs) have been found to be very popular and effective for protecting metallic units in petroleum refining, oil and gas production, exploration, the chemical industry, water treatment plants, etc. CIs are chemical substances added to corrosive environments to reduce or impede corrosion rates [5]. CIs form a protective layer on the surface of the metal to keep aggressive electrolytes from coming into contact with the metal surface. As inorganic inhibitors have already been denoted to be environmentally harmful, organic inhibitors have recently gained widespread popularity [4,19]. These inhibitors get adsorbed onto the metal surface by either an electrostatic attraction (physical adsorption or physisorption) or a coordinate covalent bond (chemical adsorption or chemisorption) when inhibitor molecules containing lone pairs of electrons donate electrons to the vacant *d-*orbitals of the metal. On the other hand, the metal can sometimes donate electrons (retro-donation) to the inhibitor molecules to form that protective layer. As a consequence, organic compounds containing heteroatoms (O, N, P, and S), polar functional groups (−COOH, −OH, −NH_2_, −CN, −C=O, −NO_2_), conjugated bonds, aromatic rings, π-systems, etc., are known to provide the best corrosion protection.

**Figure 1:**
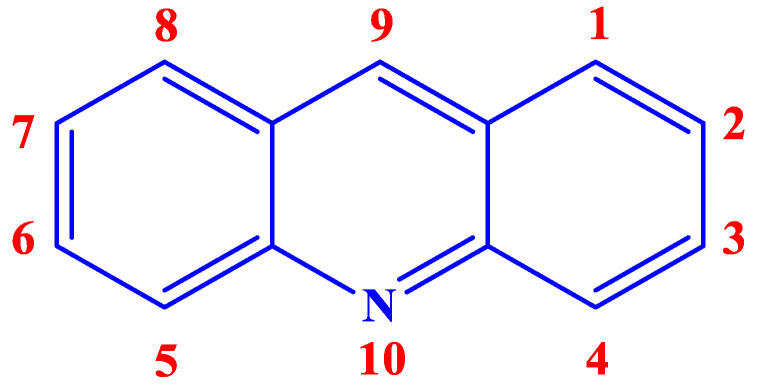
Chemical structure and numeration of acridine.

Acridine (Figure 1), an alkaloid from anthracene, also known by the names of 10-azaanthracene, dibenzopyridine, 2,3,5,6-dibenzopyridine, and its derivatives or analogs, have a great potential to be an efficient class of excellent inhibitor. Aromatic rings, π-systems, and a nitrogen atom with an available lone pair of electrons already present in the chemical structure of acridine hint at the possibility of it acting as a suitable inhibitor. Additionally, incorporating different functional groups that can undergo chemisorption or physisorption with the vacant *d-*orbitals of the metal in the acridine can make it even better as a CI. Furthermore, acridine and its analogs have been reported to have shown loads of biological activities, rendering it environmentally safe as well.

## 2. Biological Properties of Acridines

Inorganic salts and salts of heavy metals had been used historically as CIs, including molybdates, nitrates, phosphates, polyphosphates, phosphonates, sodium chromates, silicates, hydroxides, etc. [20]. However, even after showing practical efficiencies, these types of inhibitors have been restricted in their use, having been identified as toxic to human life and the environment. As a consequence, several tough regulations, including the US Occupational Safety and Health Administration (OSHA) in 1993, the Emergency Planning and Community Right to Know Act of 1986, the Chemical Hazard Assessment and Risk Management (CHARM) model adopted in the UK and other European countries, caused researchers to look for non-toxic yet efficient corrosion inhibitors. Acridine and its derivatives are found in natural plants and marine organisms, forming an important class of *N*-containing heterocycles. Due to their unique chemical and physical properties and biological activities, acridine derivatives are used across different industries. Pharmaceutically, acridine derivatives have been found to show bio-activities, such as anticancer, antitubercular, antiviral, antimalarial, antimicrobial, anti-inflammatory, antiparasitic, and fungicidal activities [21]. Some acridine derivatives, their structures, and relevant bio-activity have been included in Table 1 to show that acridine and its derivatives are being explored as environmentally friendly and efficient CIs.

## 3. Synthesis of Acridines

Acridine was isolated for the first time, from a high boiling fraction of coal tar, by Grabe and Heinrich in Germany in 1870. Due to the scarcity of quinine during World War II, an acridine-based antimalarial drug called mepacrine gained widespread popularity. However, current antibacterial therapy like penicillin and sulfonamide suppress mepacrine. Recently, an across-the-board increase in drug resistance has pushed scientists and researchers to look for alternatives, giving acridine research a boost once again, possibly making acridines suitable for use across other platforms, such as corrosion protection [31]. Although several methods are available for synthesizing acridines, some important, popular, simple, and named synthesis processes of acridines are outlined below.

**Scheme 1:**
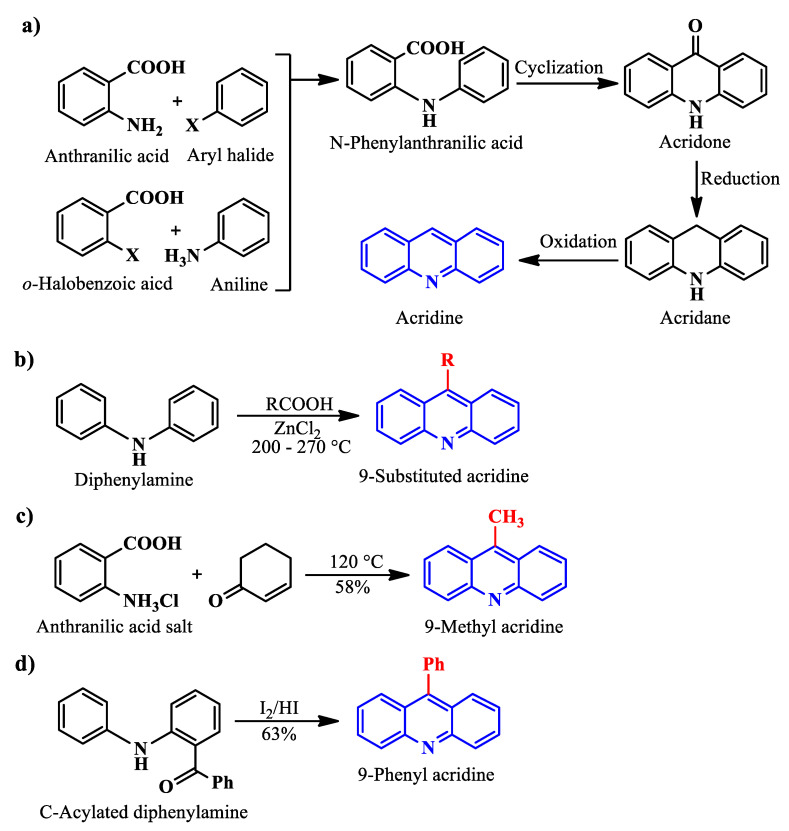
Acridine and substituted acridine synthesis using Ullman (**a**), Bernthsen (**b**), Friedlander (**c**) synthesis, and from C-acylated diphenylamine (**d**).

Ullmann synthesis [21], a prevalent method to produce acridines, involves condensing a primary amine with an aromatic aldehyde/carboxylic acid in the presence of a strong mineral acid (H_2_SO_4_/HCl), followed by a cyclization step to produce acridone. After that, two additional steps of reduction and dehydrogenation afford acridine (Scheme 1a). 9-substituted acridines can be produced by exploiting Bernthsen synthesis [32], which involves reacting diphenylamine with a carboxylic acid in the presence of zinc chloride (Scheme 1b). On the other hand, 9-methyl acridine can be afforded via Friedlander synthesis [33] when the salt of anthranilic acid is treated with 2-cyclohexenone at 120 °C (Scheme 1c). C-Acylated diphenylamine, a little variation of the Bernthsen synthesis reactant, can produce 9-phenyl acridines when treated with I_2_/HI (Scheme 1d).

## 4. Acridines as Corrosion Inhibitors

The use of acridines as corrosion inhibitors dates back to 1975 when Patel and Patel [34] used the weight loss (WL) technique to examine the anticorrosion properties of acridine and analogous phenazine and xanthine toward the corrosion of 63/67 brass in 2.0 N nitric acid (HNO_3_) solution. The metal specimens were immersed in the HNO_3_ solution for 20 min at 25 °C. Among the three inhibitors, acridine showed the best inhibition efficiency (IE), with an IE of 94% achieved for an inhibitor concentration of 0.004% and a maximum IE of 99.0% obtained for a 0.02% inhibitor concentration. Later on, Sheth and Char [35] studied the inhibitive properties of acridine, nicotinic acid, thiourea, strychnine, and quinine hydrochloride toward aircraft aluminum–zinc alloy in 0.5 N hydrochloric acid (HCl) solution. The first three inhibitors, specially acridine, performed well. Acridine showed IE close to 100% for concentrations as low as 0.5 g/L. The authors also performed polarization studies to conclude that the inhibitors mainly acted as cathodic inhibitors, as the corrosion potential became more negative with the addition of inhibitors. In the 80s, Talati and Gandhi [36], in a bid to promote the use of organic dyes as CIs, utilized acridine orange along with several other organic dyes for the protection of Al–4Cu alloy (B26S) in HCl solutions used for the pickling of aluminum or the chemical or electrochemical etching of aluminum foils. However, the IE shown by acridine orange turned out to be very unsatisfactory, with only approximately 20% efficiency offered for an inhibitor concentration of 1.0% in 0.5 M HCl at 30 °C. Even though any satisfactory conclusion regarding this low efficiency was not offered, steric effects due to the presence of different substituent groups in acridine orange might have been responsible for this. On the other hand, another study [37] by the same authors showed that, when acridine and some other *N*-heterocycle-based CIs were used at even a lower concentration of 0.5% for the protection of the same alloy in even a higher concentration of 1.0 M HCl, acridine showed an impressive IE of 99.0%, with acridine being able to confer 99–100% IE up to 5.0 M concentration of HCl. A significant shift in the cathodic Tafel slopes ($\beta_{c}$) for inhibited (with 0.05 M inhibitors) acid solution compared to the blank 0.5 M HCl solution convinced the authors to confer that the inhibitors were of a cathodic nature. Since the plot of log(θ)/(1−θ) vs. log (inhibitor concentration) showed a straight line, the inhibitors were believed to have undergone adsorption via the Langmuir adsorption isotherm. In the late 80s, Subramanyam et al. [38] studied the effects of two quinolines and acridine on the corrosion of copper in 0.1 M H_2_SO_4_ at 303 K. Acridine, as well as both quinolines, showed very high IE at a concentration of 1 × 10^−3^ M, with acridine offering 95.3% efficiency. The corrosion potential ($E_{corr}$) drifting toward a more noble direction on the addition of the inhibitors indicated that they acted as anodic-type inhibitors.

Das [39] evaluated the anticorrosion performance of acridine, *p*-amino benzaldehyde (PABD), and diethanol amine toward carbon steel in citric acid. Acridine and PABD showed better performance than diethanol amine, with both acridine and PABD showing 85.0% IE for the concentration of 1.43 mM and 333 K. A thermodynamic study revealing the Gibbs free energy of adsorption ($\Delta G_{ads}^{{}^{\circ}}$) and entropy of adsorption ($\Delta S_{ads}$) to be negative and positive, respectively, indicated that the adsorption of the inhibitors onto the carbon steel surface was spontaneous in nature. On the other hand, the heat of adsorption ($\Delta H_{ads}$) being less than 25 kJ/mol indicated that the studied inhibitors underwent physical adsorption onto the carbon steel surface. Again, the plot of log(θ)/(1−θ) vs. log (inhibitor concentration) producing straight lines with a very high correlation coefficient disclosed that the adsorption process followed the Langmuir adsorption isotherm. Zucchi et al. [40] evaluated the IE of acridine along with several other inhibitors for nickel corrosion in 0.1 M HClO_4_ using electrochemical quartz crystal microbalance (EQCM). EQCM is a very sensitive technique that can determine the mass change in systems wherein the rate of corrosion is very low. Unfortunately, acridine showed an IE of ~53.0% at a concentration of 10^−3^ M, with the negative sign implying that acridine stimulated the corrosion rate instead of suppressing it. Even though no reasoning behind this terribly poor performance by acridine was offered, acridine undergoing localized adsorption onto the nickel surface might have caused this.

During industrial hot-dip coatings, diluted acid solutions are sometimes used to dissolve the oxide layer formed on the hot-dip coating so that the adhesion between the oxide layer and an additional organic coating is enhanced to provide good corrosion protection. However, acid solutions are aggressive toward metal specimens, and inhibitors are required to prevent excessive acid consumption. Even though Cr (VI)-based inhibitors used to be a hot favorite for this purpose, their use, as we discussed earlier, has been ruled out due to their toxic and carcinogenic nature. To that end, Ju et al. [41] tested the IE of acridine toward hot-dip Zn, 5% Al–Zn, and 55% Al–Zn-coated low carbon steel sheets in 0.1 M, 0.2 M, and 0.5 M HCl solutions, respectively. Acridine, at a concentration of 10^−2^ M, imparted an IE of 99.3%, 93.7%, and 97.2% to Zn, 5% Al–Zn, and 55.0% Al–Zn, respectively, measured via the WL technique at 298 K. IE measured via electrochemical impedance spectroscopy (EIS) were closely match with the ones disclosed by the WL technique, indicating a good agreement between the results. $\Delta G_{ads}^{{}^{\circ}}$ values were found to be negative for the adsorption of acridine onto all three different types of hot-dip coatings, implying that the adsorption process was spontaneous. A short investigation into density-functional-theory-based quantum chemical calculation (DFT–QCC) revealed that the adsorption of acridine onto the Zn surface happens via interaction between Zn atoms and cyclic molecular π-orbital. Zn has an electron configuration in the form of [Ar] *3d*^10^ *4s*^2^. It is believed that acridine’s highest occupied molecular orbital (HOMO) interacts with the unfilled *4p* orbitals of Zn via coordinate covalent bonding (chemisorption). In contrast, the filled *4s* orbital of the latter interacts with the former’s lowest unoccupied molecular orbital (LUMO) via retro-donation. A large contact area with a nearly 0° angle, owing to acridine’s planar structure, can be facilitated between acridine and the metal specimen, making acridine provide enormous corrosion protection in most cases.

Hamzi et al. [42] studied the role of acridin-9(10*H*)-one as a corrosion inhibitor for the protection of mild steel in 1 M HCl. The inhibitor showed excellent IE of 98.3% via the WL method for a concentration of 10^−3^ M. A PDP study revealed that the inhibitor acted mainly by blocking the anodic dissolution of the metal surface. The $\Delta G_{ads}^{{}^{\circ}}$ value was found to be −44.03 kJ/mol, indicating that the adsorption process was spontaneous and also confirming that the inhibitor underwent chemisorption onto the mild steel surface. The inhibitor was found to follow the Langmuir adsorption isotherm model, with a very strong adsorption coefficient $\left(K_{ads}\right)$ value of 52.9 × 10^4^ M^−1^. Hamzi et al. [43] also studied the anticorrosion properties of 2-methylacridin-9(10*H*)-one (MAO) toward carbon steel in 1 M HCl. At a concentration of 10^−3^ M, MAO showed a very good IE of 95.6%. A PDP study revealed that MAO acted as a mixed-type inhibitor, as there was no significant shift in the $E_{corr}$ values. The adsorption of MAO onto the mild steel surface happened via a mixed chemisorption and physisorption process and the absorption process followed both Langmuir and Flory–Huggins adsorption isotherms. Jaralla and Al-Darbi [44] formulated a very effective industrial cleaning solution containing acridine orange inhibitor, a surfactant, and chelating and complexing agents. The inhibitor package was found to block both the anodic dissolution of metal and the evolution of molecular hydrogen, as the inhibitor showed a very strong tendency for protonation in acidic solution. A reasonably good IE of 92.0% was achieved for 80 ppm of the formulation in 5% HCl at 50 °C under 1000 rpm rotation speed.

**Figure 2:**
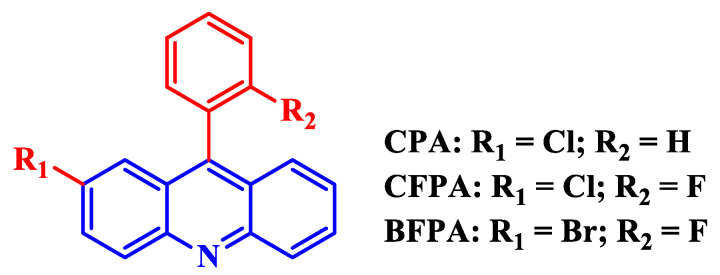
Molecular structures of halogen-substituted 9-phenyl acridines.

Salghi et al. [45] studied the anticorrosion performance of 2,10-dimethylacridin-9(10H)-one (DMA) toward C38 steel in 0.5 M H_2_SO_4_. The inhibitor, at a concentration of 10^−3^ M, was found to impart an IE of 93.6%, 92.2%, and 91.7% measured via WL, potentiodynamic polarization (PDP), and EIS techniques, respectively. It was noteworthy to mention that an inhibitor can be termed anodic or cathodic if it causes the $E_{corr}$ value to shift by more than 85 mV in the respective direction with respect to free corrosion potential, i.e., corrosion potential for the uninhibited solution. Otherwise, the inhibitor would be termed mixed-type. A PDP study revealed that DMA acted as a mixed-type inhibitor. The decreasing double-layer capacitance $\left(C_{dl}\right)$ and increasing charge transfer resistance ($R_{ct}$) values, measured via EIS, with increasing inhibitor concentration, implied a gradual replacement of water molecules by the adsorption of DMA molecules onto the C38 steel surface. It is also well known that, when the value of $\Delta G_{ads}^{{}^{\circ}}$ is −20 kJ/mol or less negative, the inhibitor is said to have undergone physisorption. On the other hand, when the value of $\Delta G_{ads}^{{}^{\circ}}$ is −40 kJ/mol or more negative, the inhibitor is then believed to be undergoing chemisorption [46]. In that context, DMA was found to be chemisorbed onto the C38 steel surface and to follow the Langmuir adsorption isotherm. As mild steel has uses across many industries, it is no wonder that they are susceptible to corrosion during industrial acid descaling, pickling, and oil well acidizing processes. Zhang et al. [47] synthesized and studied a set of halogen-substituted phenyl acridines (Figure 2) as highly effective CIs of mild steel in a 1 M HCl acidic medium. The authors envisioned that the incorporation of halogen atoms would cause the ring electrons to be drawn somewhat toward them via the inductive effect and make the non-bonding electrons on halogen atoms more available for donation to the empty orbitals of the metal atoms. As such, 2-cholor-9-phenylacridine (CPA), 2-chloro-9(2-fluorophenyl)acridine (CFPA), and 2-bromo-9(2-fluorophenyl)acridine (BFPA) were tested via WL, PDP, and EIS techniques. All three inhibitors generally showed more than 90% IE at a concentration of 0.40 mM measured via all techniques. However, the observed trend of IE was BFPA > CFPA > CPA, with BFPA showing more than 98.0% IE. The energy difference between HOMO and LUMO, ΔE, measured via DFT–QCC, is a good indicator of how good an inhibitor is. Usually, the lower the value of ΔE, the better the CI. The ΔE values of the inhibitors under discussion were found to follow the order: ΔE_BFPA_ < ΔE_CFPA_ < ΔE_CPA_, indicating that the experimental finding of BFPA being the best was valid. Additionally, the ΔE values of the protonated forms of inhibitors were smaller than those of neutral ones, meaning that the protonated forms of the inhibitors are better candidate for adsorption, as there is always a good chance of forming onium-type ions in a highly acidic medium of 1 M HCl. A PDP study revealed that the inhibitors were of mixed-type with cathodic predominance, and a thermodynamic study showed that the inhibitors followed the Langmuir adsorption isotherm.

Oil-well stimulation procedure in the oil industries involves pumping acid (mainly HCl) at a very high concentration (15–28%) to dissolve rock formations at high temperatures to expand the flow path and remove scale. This high concentration of acid inevitably poses a great danger to the structural integrity of the pipelines. The threat is so significant that even a complete shutdown of the oil production facility may take place to incur a substantial economic loss. To that end, Zhang et al. [48] designed and synthesized three 9-substituted acridines, namely 9-methyl acridine (MA), 9-carboxyacridine (CA), and 9-aminoacridine (AA), as CIs of mild steel in a highly aggressive 15% HCl acidic condition. CA, MA, and AA, at a concentration of 300 ppm, imparted an IE of 41.9%, 89.4%, and 94.2%, respectively, measured via the WL technique toward mild steel in 15% HCl at 30 °C. This is to mention that 15% HCl is a very harsh acidic condition, and achieving an efficiency of ~90.0% for even a high concentration of 300 ppm is quite noteworthy. PDP and EIS studies also confirmed the findings of the WL study, with AA being the most efficient of all measured through all three techniques. A DFT–QCC study also confirmed that the values of ΔE followed the order: ΔE_AA_ < ΔE_MA_ < ΔE_CA_. The electron fraction (ΔN) is another measure of how good an inhibitor is in a given electrolyte, with ΔN < 0 indicating electrons transferred from the metal to the inhibitor and vice versa if ΔN > 0. CA, MA, and AA were found to have ΔN values of the order of 0.26, 0.29, and 0.39, respectively, validating the experimental results once again, showing that AA is superior to the other two inhibitors in the series. The superior corrosion protection performance of AA was attributed to an amino substituent having the most negative Hammett substituent constant (σ), as it is known that the more negative the value of σ, the more the electron-donating ability of the substituent, making amino-substituted acridine more efficient than methyl– or carboxyl–substituted acridines. Finally, the molecular dynamics (MD) study showed that the adsorption of all three inhibitors onto the Fe (110) surface resulted in the interaction of AA and Fe (110) surface has the most significant binding energy ($E_{binding}$) of 791.40 kJ/mol.

**Figure 3:**
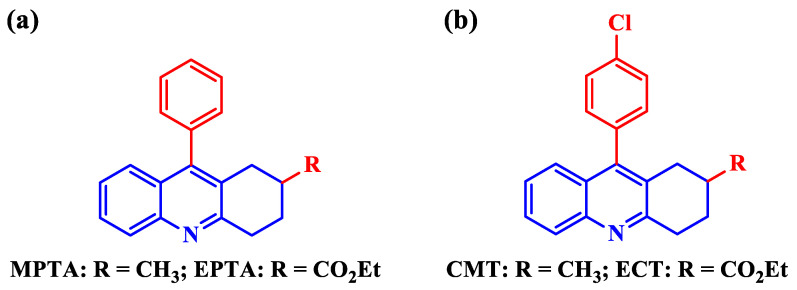
9-Phenyl substituted (**a**) 9-(4-chlorophenyl) and (**b**) tetrahydroacridines.

Zhang et al. [49] studied the anticorrosion performance of two tetrahydroacridines (Figure 3a), namely 2-methyl-9-phenyl-1,2,3,4-tetrahydroacridine (MPTA) and ethyl-9-phenyl-1,2,3,4-tetrahydroacridine-2-carboxylate (EPTA) toward X80 steel in 15% HCl medium. Because of being very stable mechanically and cost-effective at the same time, X80 steel is widely used in oil production facilities. Therefore, its degradation in harsh acidic conditions demands research studies highly. MPTA and EPTA imparted a maximum IE of 97.9% and 96.0%, respectively, at a concentration of 400 ppm. The superiority of MPTA can be attributed to −CH_3_ having a more negative value of σ than that of −CO_2_Et, meaning that −CH_3_ is a better electron-donor to make MPTA undergo much better adsorption than EPTA. The inhibitors were found to be mixed-type via a PDP study. The thermodynamic study showed that the inhibitors followed the Langmuir adsorption isotherm and underwent physisorption. Zhang et al. [50] designed and synthesized two other tetrahydroacridines (Figure 3b), namely 9-(4-chlorophenyl)-2-methyl-1,2,3,4-tetrahydroacridine (CMT) and ethyl 9-(4-chlorophenyl)-1,2,3,4-tetrahydroacridine-2-carboxylate (ECT), which are a slight variation of MPTA and EPTA, respectively, as CIs of mild steel in 1 M HCl. CMT and ECT, at a concentration of 200 ppm, imparted a maximum IE of 98.0% and 93.8%, respectively, via the WL technique. Again, the superiority of CMT to ECT is easily understandable in terms of the Hammett substituent constant. Both inhibitors were found to be of mixed type with cathodic predominance.

Cooling systems and desalination plants use seawater regularly. As seawater comprises several aggressive electrolytes in the form of sulfate ions, carbonate ions, organic acids, and most importantly, chloride ions, metallic components of the cooling systems, and desalination plants are susceptible to severe corrosion. Zhang et al. [51] developed self-assembled monolayers of 9,10-dimethylacridinium iodide (DMAI) and 9-phenyl-10-methylacridinium iodide (PMAI) onto mild steel specimens for corrosion protection against seawater. PMAI and DMAI, at a concentration of 300 mg/L, provided up to 92.3% and 89.5% IE, respectively, based on the PDP study. While PMAI having a smaller ΔE value and greater ΔN values validated the experimental finding, it is understood that the presence of a phenyl-substituent in PMAI made it more electron-rich through the resonance effect, thus making PMAI undergo better adsorption onto mild steel specimen. A surface morphology study via scanning electron microscopy (SEM) showed that the surface of bare steel in seawater was severely corroded with a very rough surface. On the other hand, DMAI- and PMAI-coated mild steel surfaces were found to be much flatter and smoother in comparison to that of the bare-coated one. Zhang et al. [52] used an environment-friendly scaling-corrosion inhibitor composed of 10-methylacridinium iodide and sodium citrate (MAI–SC) for the corrosion protection of mild steel in seawater. MAI–SC was found to be a mixed-type, with anodic predominance, i.e., mainly suppressing the anodic dissolution of the metal. While MAI–SC showed an excellent 98.3% scale IE, it also imparted a reasonably good corrosion IE of 92.7%, with the mass ratio of MAI and SC being 1:2 for corrosion inhibition. However, it has been observed many times that some nitrogen-containing heterocyclic compounds are not protecting metal specimens in neutral/nearly neutral solutions, as those inhibitors are not soluble in those conditions in the first place. One strategy to avoid this problem is to create hybrid inhibitors containing organic and inorganic moieties. Zhang et al. [53] developed an acriflavine–Zn^2+^ complex (AF–Zn^2+^) to protect mild steel in seawater conditions. AF and Zn^2+^ at a ratio of 1:1 provided the best IE. While AF alone imparted an IE of 72.5% at a concentration of 120 ppm, AF–Zn^2+^ (120/120) provided an increased IE of 94.6%. The PDP study revealed that AF acted as a mixed-inhibitor, but AF–Zn^2+^ behaved as an anodic inhibitor. The ΔN for AF and AF–Zn^2+^ was found to be −0.037 and −0.049, respectively, indicating that both inhibitors were electron acceptors from the metal surface via back donation, with the latter being stronger electron acceptors, thus providing more efficiency. SEM and XPS studies provided strong evidence of the formation of protective films on the mild steel surface. The XPS study showed that the protective film was composed mainly of [Fe (III), Zn (II)–AF] complex, Zn(OH)_2_, and some oxides and hydroxides of iron (III).

**Figure 4:**
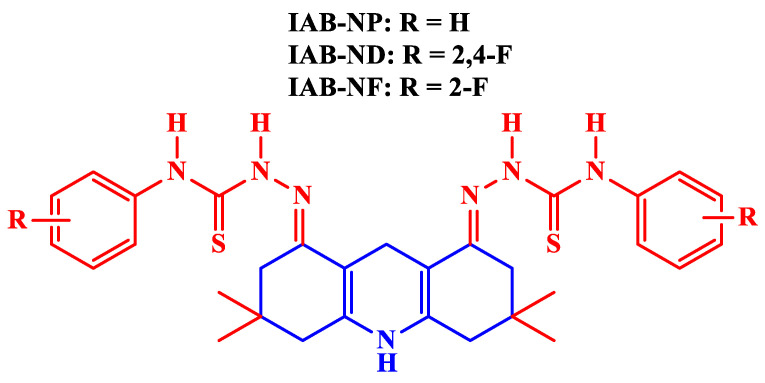
Acridine-based thiosemicarbazones as corrosion inhibitors.

Akpan et al. [54] used three acridine-based thiosemicarbazones (Figure 4), namely (2*E*,2’*E*)-2,2’-(3,3,6,6-tetramethyl-9-phenyl-3,4,6,7-tetrahydroacridine-1,8(2*H*,5*H*,9*H*,10*H*)-diylidene bis(*N*-phenylhydrazinecarbothioamide) (IAB-NP), (2E,2’E)-2,2’-(3,3,6,6-tetramethyl-9-phenyl-3,4,6,7-tetrahydroacridine-1,8(2*H*,5*H*,9*H*,10*H*)-diylidene)bis(*N*-(2,4-difluorophenyl) hydrazinecarbothioamide) (IAB-ND) and (2E,2’E)-2,2’-(3,3,6,6-tetramethyl-9-phenyl-3,4,6,7-tetrahydroacridine-1,8(2*H*,5*H*,9*H*,10*H*)-diylidene)bis(*N*-(2-fluorophenyl) hydrazinecarbothioamide) (IAB-NF) as CIs of mild steel in 1 M HCl. It is worth mentioning that these acridine-based thiosemicarbazones initially developed by Isaac et al. [55] were used as chemosensors for the selective recognition of fluoride ions. As fluoride ion is regularly used in making toothpaste and the treatment of osteoporosis, it is a biologically very important anion, which is explored as an environment-friendly scaffold for the anion-sensing process. The inhibitors were observed to be more efficient with increasing concentration. However, at a concentration of 1.5 × 10^−5^ M, IAB-NP, IAB-ND, and IAB-NF were found to impart a maximum IE of 85.3%, 87.5%, and 90.5%%, respectively. A PDP study revealed that the inhibitors acted as mixed-type. A thermodynamic study confirmed that the inhibitors followed the Langmuir adsorption isotherm and underwent mixed-type adsorption. A DFT–QCC study showed that IAB-ND and IAB-NF had ΔN values more negative and electronegativity (χ) values greater than IAB-NP, indicating that IAB-ND and IAB-NF were more disposed to back-donation than IAB-NP, which led to IAB-ND and IAB-NF undergo much better adsorption. Additionally, IAB-ND and IAB-NF were found to have more dipole moments than IAB-NP, meaning that IAB-ND and IAB-NF had much better dipole–dipole interactions with the polarized mild steel surface.

In contrast to the study by Talati and Gandhi [36] that saw acridine orange imparting very low IE to Al–4Cu alloy (B26S) in HCl solutions, Sahin [56] used acridine orange very successfully for the corrosion protection of copper in 0.5 M H_2_SO_4_. As copper possesses extraordinary thermal, mechanical, and electrical properties, they have widespread uses across many fields, such as bio-sensors, conductive circuit boards, microelectronics, heat exchangers, pumps, electrical switches, etc. However, in order to remove corrosion products, rust, and undesired oxides, pickling operations using sulfuric acid are often carried out, which, unfortunately, damage copper and its alloys in the process. Sahin’s study showed that acridine orange could impart very high IE at very low concentrations, with 0.01 mM of acridine orange imparting an extremely high IE of 99.1% and a maximum IE of 99.4% being achieved for 1 mM inhibitor concentration. A PDP study showed that the compound acted as a mixed-inhibitor with cathodic predominance. A negative value (−27.7 kJ/mol) of $\Delta G_{ads}^{{}^{\circ}}$ and a very high value (1.298 × 10^3^ M^−1^) of the adsorption equilibrium constant showed that the adsorption was spontaneous and very strong. Parveen et al. [57] used different acridones as acetylcholinesterage (AChE) inhibitors against various bacterial strains. 2-nitroacridone showed an IC_50_ value of 0.45 μM, which was very much comparable with the standard (Tacrine). Aslam et al. [58] used 2-nitroacridone as the CI of low-carbon steel in 1 M HCl solution. A range of inhibitor concentrations (0.001–10 mM) and temperatures (25–65 °C) was tested to find out the optimum inhibitor concentration and operating temperature. 2-Nitroacridone was found to act best and to impart a maximum IE of 94.9% at a concentration of 5 mM at 25 °C. A PDP study showed that the inhibitor served as a mixed-type, with anodic predominance.

## 5. Inhibition Mechanism of Acridines

**Figure 5:**
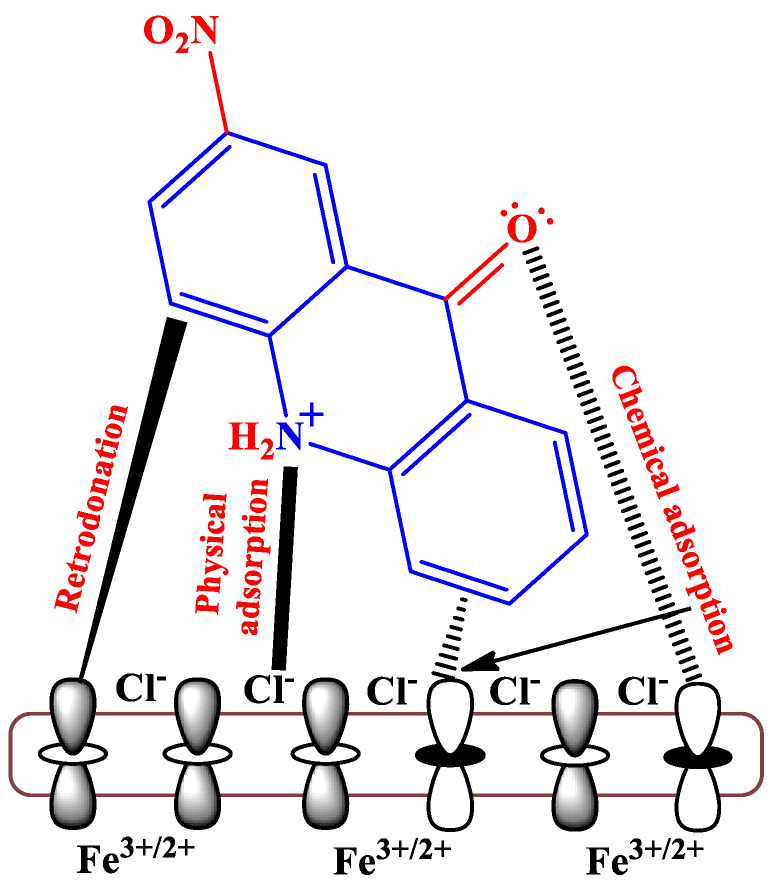
Protonated form of 2-nitroacridone undergoing adsorption onto the metal specimen.

As we have observed throughout the discussion, acridine performed well in almost all harsh acidic conditions, even in a 15% HCl medium. Acridine, which is basically an *N*-heterocyclic compound, possesses multiple aromatic rings, conjugated π-bonds, π-centers, and heteroatoms with lone pairs of electrons, imparts great IE to metal specimens [59]. Additionally, acridine’s planar structure helps it thicken a metal surface around aggressive electrolytes. Figure 5 shows multiple interactions between a protonated form of 2-nitroacridone and a metal surface. Note that the inhibitor has been shown in a tilted position to visualize different interactions. In an uninhibited HCl solution, water molecules carrying H^+^ and Cl^−^ ions are adsorbed onto the metal surface, bringing destruction to metallic components in the forms of molecular hydrogen evolution as a cathodic-half reaction and the dissolution of metal components as Fe^2+^ or Fe^3+^ as an anodic-half reaction [60]. In acidic solutions, *N*-heterocyclic compounds undergo protonation in most cases, allowing them to have electrostatic interaction (physical adsorption or physisorption) with Cl^−^ ions latched onto the metal surface.

On the other hand, lone pair of electrons on heteroatoms and/or π-electrons from aromatic rings or conjugated bonds can be donated to empty *d*-orbitals of metal atoms to cause n→d and π→d chemisorption, respectively. However, sometimes electrons from the filled *d*-orbitals of metal atoms can be donated to π-antibonding orbitals to cause d→π^*^ retro-donation interaction [5]. Furthermore, it is noteworthy that the presence of some hydrophobic tails in inhibitors helps expel the water molecules adsorbed onto metal surfaces.

## 6. Conclusions and Future Outlook

In this review, we have covered scores of acridine- and acridine-based CIs for the protection of metals and metal alloys in different aggressive corrosive conditions. The fact that acridine has a planar aromatic system, multiple π-bonds, and a nitrogen atom with an available lone pair of electrons for interaction with metal surfaces pose acridines and substituted acridines as a very efficient class of inhibitors. Furthermore, as we laid out earlier in the discussion, acridines’ pharmacological activities make them environmentally benign, as the current focus of corrosion inhibitor industries is on finding inhibitors that are not only efficient but also eco-friendly. Even though the discovery of acridine dates back to as early as the late nineteenth century and the first uses of acridines as CIs to around the mid-twentieth century, acridine and its analogs have never really received as much attention as they deserve as an up-and-coming class of inhibitors. To the best of our knowledge, we have discussed all the literature available on acridines and substituted acridines used in an experimental setup to protect metals and alloys. However, it is worth mentioning that the availability of acridine-based CIs tested in basic conditions is scarce or not available at all, as our extensive search did not bring any such articles to our attention. This could be owing to the fact that acridine-based CIs are probably best-suited to their efficacy in a wide range of acidic media. In particular, pure acridine shows efficacies of 99.0% towards 63/67 brass in 2.0 N HNO_3_ [34], 100% towards Al–Zn alloy in 0.5 N HCl [35], 99–100% towards Al–4 Cu alloy in 5.0 M HCl [37], 95.3% towards Cu in 0.1 M H_2_SO_4_ [38], and 99.3% towards Zn in 0.1 M HCl [41] for extremely low concentrations, which are truly phenomenal and exciting results. Some acridine-based inhibitors (at a meager concentration) performed well in protecting mild steel in 1 M HCl, which is regularly used for industrial acid cleaning, acid pickling, and acid descaling processes. For illustrations, acridin-9(10*H*)-one providing 98.3% [42], 2-bromo-9(2-fluorophenyl)acridine (BFPA) imparting 98.0% [47], and 9-(4-chlorophenyl)-2-methyl-1,2,3,4-tetrahydroacridine (CMT) providing 98.0% IE [50] toward mild steel in 1 M HCl are really noteworthy. Additionally, 9-aminoacridine (300 ppm) showing 94.2% IE toward mild steel [48] and 2-methyl-9-phenyl-1,2,3,4-tetrahydroacridine (MPTA; 400 ppm) showing 97.9% IE toward X80 steel [49] in very harsh 15% HCl are remarkable. With increasing antibacterial resistance worldwide and researchers looking for better and more efficient drugs consistently, acridines have recently grabbed much attention. This, in turn, could open new vistas for applying acridine-based CIs in other electrolytic conditions, including basic ones. Actually, there are already scores of acridines and their analogs that have been synthesized but not explored as CIs yet. Rupar et al. [61], Prasher and Sharma [62], and Gensicka-Kowalewska et al. [21] discussed many acridines and acridine-based compounds that have been synthesized recently for their biological activities. Importantly, this review encourages corrosion enthusiasts to look into these new acridines. We hope this review will open up more scope for acridines to be used as CIs and make researchers more aware of their presence.

## Figures and Tables

**Table 1:** Several pharmaceutically important acridine derivatives and their chemical structures.

Bioactivity	Acridine Derivative	Remarks	References
Antimalarial	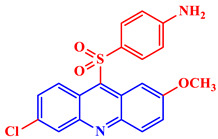 2-Methoxy-6-chloro-9-(4′-amino-phenylsulfonyl)-acridine	IC_50_ values of 14.3 and 20 μM are shown against 3D7 and W2 strains, respectively, of *Plasmodium falciparum*.	[22]
	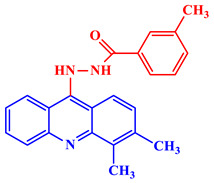 *N*′-(3,4-dimethyl-9-acridinyl)-2-hydroxybenzohydrazide	IC_50_ values of 0.5 ± 0.05 and 0.13 ± 0.03 nM are shown against human cathepsin D and *Plasmodium falciparum* plasmepsin-II, respectively.	[23]
	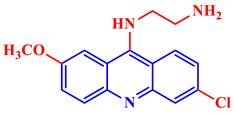 9-(*N*-ethylamino)-6-chloro-2-methoxyacridine	IC_50_ values of 42 ± 19 and 280 ± 40 nM are shown against chloroquinone-susceptible and chloroquinone-resistant *Plasmodium falciparum* strains, respectively.	[24]
Antiviral	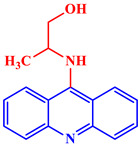 9-(*N*-2-methyl hydroxyethyl)acridine	An MIC_50_ of 5 μg/mL was enough to reduce the infection viral titer of HSV-L_2_ in an experiment by order of 6.5 to that of the control.	[25]
	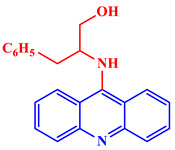 9-(*N*-2-benzyl hydroxyethyl)acridine	An MIC_50_ of 5 μg/mL was enough to reduce the infection viral titer of HSV-L_2_ in an experiment by order of 1.3 to that of the control.	[25]
Antibacterial	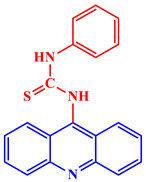 1-(9-AcridinyI)-3-phenyl-2-thiourea	At 400 μg/mL levels, the compound effectively inhibited strains of several bacterial cell lines, including *Salmonella pullorum* and *Escherichia coli*.	[26]
	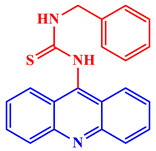 1-(9-AcridinyI)-3-benzyl-2-thiourea	At 400 μg/mL levels, the compound effectively inhibited strains of several additional bacterial cell lines.	[26]
	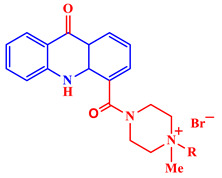 1-Methyl-1-[(5-nitrofuran-2-yl)methyl]-4-(9-oxo-9,10-dihydroacridine-4-carbonyl)piperazin-1-ium bromide	The compound showed commendable antibacterial activity against multiple test strains, including *S. aureus* and *E. coli.*	[27]
Antitumor	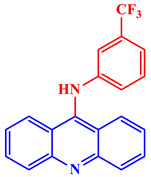 9-[(3′-Trifluoromethyl)phenylamino]acridine hydrochloride	CTC_50_ values of 31.25 and 36.25 μg/mL were shown against cervical cancer cell (*HeLa*) and lung cancer cell (*A-549*) lines, respectively.	[28]
	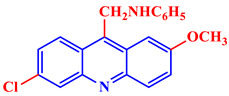 6-Chloro-2-methoxy-*N*-benzylacridin-9-amine	IC_50_ values of 0.65 and 1.40 μM were shown against K562 leukemia and hepatoma HepG-2 cell lines, respectively.	[29]
	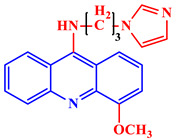 *N*-(3-(1*H*-imidazol-1-yl)propyl)-4-methoxyacridin-9-amine	Excellent anticancer activity was shown against multiple cell lines, including liver (HEP-2), breast (MCF-7), and lung (*A-549*) cell lines.	[30]
